# Crystal structure of 1-cyclo­propane­carbon­yl-3-methyl-2,6-di-*p*-tolyl­piperidin-4-one

**DOI:** 10.1107/S1600536814018546

**Published:** 2014-08-30

**Authors:** A. Kamaraj, S. Ranjith, R. Rajkumar, G. Mohanraj, K. Krishnasamy

**Affiliations:** a Department of Chemistry, Annamalai University, Annamalainagar 608 002, Chidambaram, Tamilnadu, India; b Department of Physics, SRM University, Ramapuram Campus, Chennai 600 089, India; c Post Graduate and Research Department of Botany, Pachaiyappa’s College, Chennai 600 030, Tamil Nadu, India

**Keywords:** crystal structure, cyclo­propane­carbon­yl, p-tolyl­piperidin-4-one, C—H⋯π inter­action

## Abstract

The title compound, C_24_H_27_NO_2_, crystallizes with two independent mol­ecules (*A* and *B*) in the asymmetric unit. The two mol­ecules have very similar conformations and each exhibits an intra­molecular C—H⋯π inter­action. The central piperidine rings adopt boat conformations and the *p*-tolyl rings are inclined to the mean plane of the piperidine ring by 71.21 (11) and 89.86 (12)° in mol­ecule *A* and by 68.01 (12) and 89.33 (12)° in mol­ecule *B*. The cyclopropanecarbonyl group is oriented at an angle of 68.5 (2)° with respect to the mean plane of the piperidine ring in mol­ecule *A* and 66.2 (2)° in mol­ecule *B*. In the crystal, the *A* and *B* mol­ecules are linked by C—H⋯O hydrogen bonds, enclosing *R*_2_^1^(6) ring motifs, forming ribbons running along the *a-*axis direction.

## Related literature   

For the biological activity of piperidine derivatives, see: Aridoss *et al.* (2009 ▶); Nalanishi *et al.* (1974 ▶); Michael (2001 ▶); Pinder (1992 ▶); Rubiralta *et al.* (1991 ▶). For the crystal structure of the related compound, 2,2-di­chloro-1-(4-eth­oxy­phen­yl)cyclo­propanylpiperidin-1-yl ketone, see: Sun *et al.* (2006 ▶).
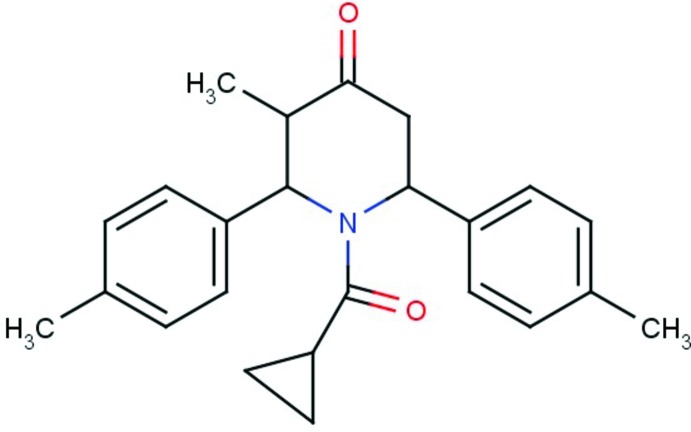


## Experimental   

### Crystal data  

C_24_H_27_NO_2_
*M* *_r_* = 361.47
Triclinic, 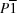

*a* = 10.494 (3) Å
*b* = 11.464 (2) Å
*c* = 17.425 (3) Å
α = 80.265 (15)°
β = 86.064 (17)°
γ = 89.877 (18)°
*V* = 2061.1 (7) Å^3^
*Z* = 4
Mo *K*α radiation
μ = 0.07 mm^−1^
*T* = 293 K
0.25 × 0.22 × 0.19 mm

### Data collection  

Bruker APEXII CCD area-detector diffractometer
Absorption correction: multi-scan (*SADABS*; Sheldrick, 1996 ▶) *T*_min_ = 0.982, *T*_max_ = 0.986
13683 measured reflections
7267 independent reflections
4028 reflections with *I* > 2σ(*I*)
*R*_int_ = 0.032

### Refinement  

*R*[*F* ^2^ > 2σ(*F* ^2^)] = 0.054
*wR*(*F* ^2^) = 0.156
*S* = 0.95
7276 reflections
493 parameters
H-atom parameters constrained
Δρ_max_ = 0.16 e Å^−3^
Δρ_min_ = −0.14 e Å^−3^

### 

Data collection: *APEX2* (Bruker, 2008 ▶); cell refinement: *SAINT* (Bruker, 2008 ▶); data reduction: *SAINT*; program(s) used to solve structure: *SHELXS97* (Sheldrick, 2008 ▶); program(s) used to refine structure: *SHELXL97* (Sheldrick, 2008 ▶); molecular graphics: *ORTEP-3 for Windows* (Farrugia, 2012 ▶); software used to prepare material for publication: *SHELXL97* and *PLATON* (Spek, 2009 ▶).

## Supplementary Material

Crystal structure: contains datablock(s) global, I. DOI: 10.1107/S1600536814018546/su2765sup1.cif

Structure factors: contains datablock(s) I. DOI: 10.1107/S1600536814018546/su2765Isup2.hkl

Supporting information file. DOI: 10.1107/S1600536814018546/su2765Isup3.cml

**Fig. 1.**
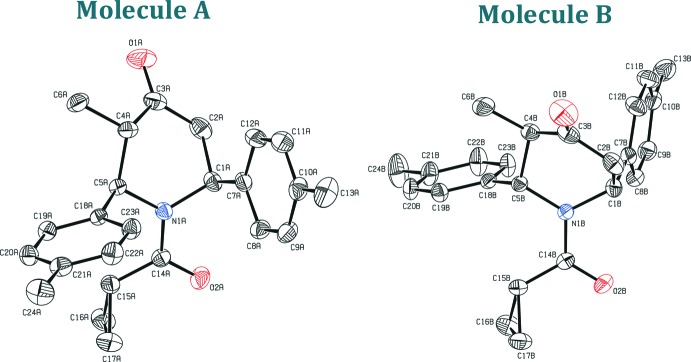
The molecular structure of the two independent molecules of the title compound, with atom labelling. Displacement ellipsoids are drawn at the 30% probability level.

. DOI: 10.1107/S1600536814018546/su2765fig1.tif
The mol­ecular structure of the two independent mol­ecules of the title compound, with atom labelling. Displacement ellipsoids are drawn at the 30% probability level.

**Fig. 2.**
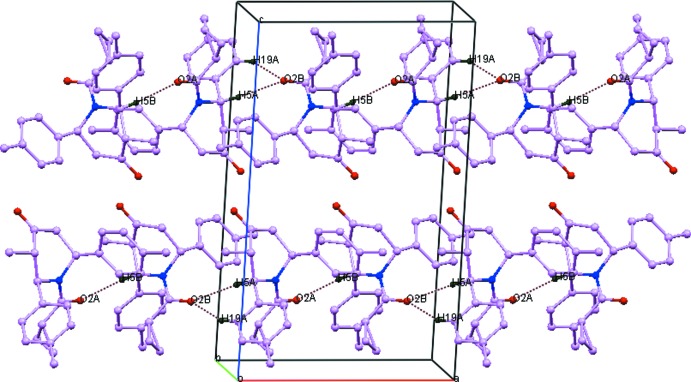
The crystal packing of the title compound, viewed along the b axis, in which hydrogen bonds form R21(6) bifurcated ring motifs forming ribbons running along a axis. For the sake of clarity, H atoms not involved in hydrogen bonds have been omitted.

Click here for additional data file.b a . DOI: 10.1107/S1600536814018546/su2765fig2.tif
The crystal packing of the title compound, viewed along the *b* axis, in which hydrogen bonds form 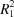
(6) bifurcated ring motifs forming ribbons running along *a* axis. For the sake of clarity, H atoms not involved in hydrogen bonds have been omitted.

CCDC reference: 922988

Additional supporting information:  crystallographic information; 3D view; checkCIF report

## Figures and Tables

**Table 1:** Hydrogen-bond geometry (Å, °) *Cg*1 and *Cg*2 are the centroids of the C7*A*–C12*A* and C7*B*–C12*B* rings, respectively.

*D*—H⋯*A*	*D*—H	H⋯*A*	*D*⋯*A*	*D*—H⋯*A*
C23*A*—H23*A*⋯*Cg*1	0.93	2.86	3.787 (3)	173
C23*B*—H23*B*⋯*Cg*2	0.93	2.85	3.777 (3)	173
C5*A*—H5*A*⋯O2*B* ^i^	0.98	2.41	3.366 (3)	166
C5*B*—H5*B*⋯O2*A*	0.98	2.42	3.381 (3)	166
C19*A*—H19*A*⋯O2*B* ^i^	0.93	2.59	3.435 (3)	151

Symmetry code: (i) 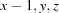.

## supplementary crystallographic information

### S1. Synthesis and crystallization

To a well stirred solution of 3-methyl-2,6-di-*p*-tolyl­piperidin-4-one (1 equiv) and tri­ethyl­amine (1 equiv) in 30 ml of dry benzene, cyclo­propane­carbonyl chloride (1 equiv) in 20 ml of benzene was added drop wise over a period of 30 min. Stirring was continued with mild heating using a magnetic stirrer. After the completion of reaction, it was poured into water and extracted with ether in three 50 ml portions. The combined ether extract was then washed well with 3% sodium bicarbonate solution and dried over anhydrous sodium sulfate. Crystal were obtained by slow evaporation of a solution of the title compound in ethanol.

### S2. Refinement

Crystal data, data collection and structure refinement details are summarized in Table 1. All H atoms were fixed geometrically and allowed to ride on their parent atoms: C—H = 0.93–0.97 Å with *U*_iso_(H) = 1.5*U*_eq_(C-methyl) and = 1.2*U*_eq_(C) for other H atoms.

### Crystal data

**Table:**

C_24_H_27_NO_2_	*Z* = 4
*M**_r_* = 361.47	*F*(000) = 776
Triclinic, *P*1	*D*_x_ = 1.165 Mg m^−^^3^
Hall symbol: -P 1	Mo *K*α radiation, λ = 0.71073 Å
*a* = 10.494 (3) Å	Cell parameters from 7267 reflections
*b* = 11.464 (2) Å	θ = 2.7–25°
*c* = 17.425 (3) Å	µ = 0.07 mm^−^^1^
α = 80.265 (15)°	*T* = 293 K
β = 86.064 (17)°	Block, white crystalline
γ = 89.877 (18)°	0.25 × 0.22 × 0.19 mm
*V* = 2061.1 (7) Å^3^	

### Data collection

**Table:**

Bruker APEXII CCD area-detector diffractometer	7267 independent reflections
Radiation source: fine-focus sealed tube	4028 reflections with *I* > 2σ(*I*)
Graphite monochromator	*R*_int_ = 0.032
ω and φ scans	θ_max_ = 25.0°, θ_min_ = 2.7°
Absorption correction: multi-scan (*SADABS*; Sheldrick, 1996)	*h* = −11→12
*T*_min_ = 0.982, *T*_max_ = 0.986	*k* = −11→13
13683 measured reflections	*l* = −20→20

### Refinement

**Table:**

Refinement on *F*^2^	Primary atom site location: structure-invariant direct methods
Least-squares matrix: full	Secondary atom site location: difference Fourier map
*R*[*F*^2^ > 2σ(*F*^2^)] = 0.054	Hydrogen site location: inferred from neighbouring sites
*wR*(*F*^2^) = 0.156	H-atom parameters constrained
*S* = 0.95	*w* = 1/[σ^2^(*F*_o_^2^) + (0.0688*P*)^2^] where *P* = (*F*_o_^2^ + 2*F*_c_^2^)/3
7276 reflections	(Δ/σ)_max_ = 0.011
493 parameters	Δρ_max_ = 0.16 e Å^−^^3^
0 restraints	Δρ_min_ = −0.14 e Å^−^^3^

### Special details

**Table:**

Geometry. All e.s.d.'s (except the e.s.d. in the dihedral angle between two l.s. planes) are estimated using the full covariance matrix. The cell e.s.d.'s are taken into account individually in the estimation of e.s.d.'s in distances, angles and torsion angles; correlations between e.s.d.'s in cell parameters are only used when they are defined by crystal symmetry. An approximate (isotropic) treatment of cell e.s.d.'s is used for estimating e.s.d.'s involving l.s. planes.
Refinement. Refinement of *F*^2^ against ALL reflections. The weighted *R*-factor *wR* and goodness of fit *S* are based on *F*^2^, conventional *R*-factors *R* are based on *F*, with *F* set to zero for negative *F*^2^. The threshold expression of *F*^2^ > σ(*F*^2^) is used only for calculating *R*-factors(gt) *etc*. and is not relevant to the choice of reflections for refinement. *R*-factors based on *F*^2^ are statistically about twice as large as those based on *F*, and *R*- factors based on ALL data will be even larger.

### Fractional atomic coordinates and isotropic or equivalent isotropic displacement parameters (Å^2^)

**Table:**

	*x*	*y*	*z*	*U*_iso_*/*U*_eq_	
C1A	0.2419 (2)	0.2299 (2)	0.33693 (13)	0.0591 (6)	
H1A	0.2913	0.3040	0.3274	0.071*	
C1B	0.7385 (2)	0.3525 (2)	0.34051 (14)	0.0598 (6)	
H1B	0.7873	0.2822	0.3321	0.072*	
C2A	0.1458 (2)	0.2385 (2)	0.40535 (15)	0.0755 (8)	
H2A1	0.1897	0.2236	0.4532	0.091*	
H2A2	0.1137	0.3188	0.3996	0.091*	
C2B	0.6410 (2)	0.3116 (2)	0.40856 (15)	0.0755 (8)	
H2B1	0.6082	0.2344	0.4035	0.091*	
H2B2	0.6840	0.3025	0.4567	0.091*	
C3A	0.0349 (3)	0.1549 (2)	0.41292 (14)	0.0699 (8)	
C3B	0.5316 (3)	0.3931 (3)	0.41428 (15)	0.0722 (8)	
C4A	0.0335 (2)	0.0760 (2)	0.35270 (13)	0.0553 (6)	
H4A	0.1021	0.0185	0.3630	0.066*	
C4B	0.5318 (2)	0.5027 (2)	0.35287 (13)	0.0563 (6)	
H4B	0.6010	0.5542	0.3628	0.068*	
C5A	0.06279 (19)	0.14576 (18)	0.27003 (12)	0.0459 (5)	
H5A	−0.0128	0.1929	0.2559	0.055*	
C5B	0.56099 (18)	0.47286 (19)	0.27085 (12)	0.0475 (5)	
H5B	0.4848	0.4340	0.2570	0.057*	
C6A	−0.0910 (2)	0.0064 (2)	0.35850 (16)	0.0757 (8)	
H6A1	−0.1070	−0.0359	0.4107	0.114*	
H6A2	−0.0847	−0.0487	0.3227	0.114*	
H6A3	−0.1599	0.0599	0.3458	0.114*	
C6B	0.4085 (2)	0.5713 (3)	0.35773 (16)	0.0816 (9)	
H6B1	0.3384	0.5233	0.3482	0.122*	
H6B2	0.4142	0.6421	0.3193	0.122*	
H6B3	0.3950	0.5919	0.4088	0.122*	
C7A	0.3389 (2)	0.1305 (2)	0.34870 (14)	0.0571 (6)	
C7B	0.8357 (2)	0.4449 (2)	0.35131 (13)	0.0555 (6)	
C8A	0.4362 (2)	0.1284 (2)	0.29136 (15)	0.0654 (7)	
H8A	0.4416	0.1877	0.2477	0.078*	
C8B	0.9348 (2)	0.4739 (2)	0.29481 (14)	0.0619 (7)	
H8B	0.9410	0.4348	0.2521	0.074*	
C9A	0.5255 (2)	0.0395 (2)	0.29806 (17)	0.0743 (8)	
H9A	0.5906	0.0411	0.2589	0.089*	
C9B	1.0245 (2)	0.5594 (2)	0.30002 (16)	0.0679 (7)	
H9B	1.0901	0.5764	0.2610	0.082*	
C10A	0.5215 (2)	−0.0512 (2)	0.36060 (18)	0.0731 (8)	
C10B	1.0187 (2)	0.6200 (2)	0.36181 (17)	0.0679 (7)	
C11A	0.4260 (3)	−0.0472 (3)	0.41769 (17)	0.0862 (9)	
H11A	0.4210	−0.1066	0.4614	0.103*	
C11B	0.9221 (3)	0.5888 (3)	0.41889 (16)	0.0809 (8)	
H11B	0.9166	0.6267	0.4621	0.097*	
C12A	0.3370 (3)	0.0417 (3)	0.41257 (15)	0.0783 (8)	
H12A	0.2745	0.0416	0.4531	0.094*	
C12B	0.8330 (2)	0.5025 (3)	0.41386 (15)	0.0769 (8)	
H12B	0.7696	0.4833	0.4540	0.092*	
C13A	0.6162 (3)	−0.1515 (3)	0.3662 (2)	0.1086 (11)	
H13A	0.5824	−0.2152	0.3441	0.163*	
H13B	0.6309	−0.1791	0.4200	0.163*	
H13C	0.6952	−0.1239	0.3380	0.163*	
C13B	1.1129 (2)	0.7172 (3)	0.3665 (2)	0.0964 (10)	
H13D	1.0715	0.7924	0.3563	0.145*	
H13E	1.1832	0.7150	0.3285	0.145*	
H13F	1.1440	0.7060	0.4178	0.145*	
C14A	0.1987 (2)	0.3155 (2)	0.20440 (14)	0.0529 (6)	
C14B	0.6965 (2)	0.3324 (2)	0.20803 (14)	0.0531 (6)	
C15A	0.1157 (2)	0.3300 (2)	0.13864 (14)	0.0601 (6)	
H15A	0.0375	0.2817	0.1455	0.072*	
C15B	0.6111 (2)	0.3498 (2)	0.14333 (13)	0.0573 (6)	
H15B	0.5313	0.3920	0.1514	0.069*	
C16A	0.1098 (3)	0.4497 (2)	0.09105 (16)	0.0867 (9)	
H16A	0.0288	0.4746	0.0704	0.104*	
H16B	0.1598	0.5123	0.1060	0.104*	
C16B	0.6711 (3)	0.3653 (3)	0.06201 (15)	0.0875 (9)	
H16C	0.6293	0.4175	0.0218	0.105*	
H16D	0.7636	0.3660	0.0553	0.105*	
C17A	0.1782 (3)	0.3580 (3)	0.05831 (16)	0.0987 (10)	
H17A	0.2707	0.3633	0.0529	0.118*	
H17B	0.1397	0.3256	0.0173	0.118*	
C17B	0.6075 (3)	0.2534 (2)	0.09552 (15)	0.0814 (8)	
H17C	0.6608	0.1849	0.1096	0.098*	
H17D	0.5264	0.2363	0.0760	0.098*	
C18A	0.08307 (19)	0.06132 (18)	0.21231 (13)	0.0464 (5)	
C18B	0.58476 (18)	0.58418 (18)	0.21128 (13)	0.0464 (5)	
C19A	−0.0090 (2)	0.0473 (2)	0.16252 (14)	0.0565 (6)	
H19A	−0.0820	0.0934	0.1621	0.068*	
C19B	0.4943 (2)	0.6257 (2)	0.16086 (14)	0.0593 (6)	
H19B	0.4181	0.5838	0.1626	0.071*	
C20A	0.0054 (2)	−0.0349 (2)	0.11287 (14)	0.0651 (7)	
H20A	−0.0583	−0.0424	0.0794	0.078*	
C20B	0.5143 (2)	0.7287 (2)	0.10750 (15)	0.0689 (7)	
H20B	0.4511	0.7546	0.0739	0.083*	
C21A	0.1108 (3)	−0.1056 (2)	0.11164 (15)	0.0662 (7)	
C21B	0.6248 (2)	0.7937 (2)	0.10268 (17)	0.0757 (8)	
C22A	0.2031 (2)	−0.0902 (2)	0.16063 (16)	0.0735 (8)	
H22A	0.2764	−0.1359	0.1605	0.088*	
C22B	0.7141 (2)	0.7521 (2)	0.15385 (18)	0.0873 (9)	
H22B	0.7899	0.7945	0.1527	0.105*	
C23A	0.1904 (2)	−0.0082 (2)	0.21062 (14)	0.0631 (7)	
H23A	0.2549	0.0000	0.2434	0.076*	
C23B	0.6951 (2)	0.6490 (2)	0.20716 (15)	0.0672 (7)	
H23B	0.7582	0.6232	0.2408	0.081*	
C24A	0.1234 (3)	−0.1974 (3)	0.05885 (18)	0.1032 (11)	
H24A	0.0436	−0.2389	0.0607	0.155*	
H24B	0.1889	−0.2524	0.0760	0.155*	
H24C	0.1457	−0.1591	0.0063	0.155*	
C24B	0.6486 (3)	0.9056 (3)	0.0430 (2)	0.1278 (14)	
H24D	0.5799	0.9162	0.0088	0.192*	
H24E	0.7277	0.8988	0.0130	0.192*	
H24F	0.6531	0.9724	0.0693	0.192*	
N1A	0.17108 (15)	0.22916 (15)	0.26704 (10)	0.0496 (5)	
N1B	0.66769 (15)	0.38776 (15)	0.27005 (10)	0.0493 (5)	
O1A	−0.0479 (2)	0.1532 (2)	0.46437 (12)	0.1125 (8)	
O1B	0.4475 (2)	0.3708 (2)	0.46545 (12)	0.1167 (8)	
O2A	0.29081 (15)	0.38296 (15)	0.20209 (11)	0.0766 (5)	
O2B	0.78989 (15)	0.26785 (16)	0.20523 (10)	0.0792 (6)	

### Atomic displacement parameters (Å^2^)

**Table:**

	*U*^11^	*U*^22^	*U*^33^	*U*^12^	*U*^13^	*U*^23^
C1A	0.0691 (15)	0.0556 (15)	0.0546 (16)	−0.0053 (12)	−0.0155 (12)	−0.0106 (12)
C1B	0.0675 (15)	0.0546 (15)	0.0560 (16)	0.0074 (12)	−0.0123 (12)	−0.0021 (12)
C2A	0.0923 (19)	0.080 (2)	0.0613 (18)	0.0145 (16)	−0.0129 (15)	−0.0281 (15)
C2B	0.0910 (19)	0.0755 (19)	0.0538 (17)	−0.0197 (15)	−0.0091 (14)	0.0088 (14)
C3A	0.0815 (18)	0.0777 (19)	0.0480 (16)	0.0174 (15)	0.0027 (14)	−0.0064 (14)
C3B	0.0752 (18)	0.091 (2)	0.0496 (17)	−0.0203 (15)	0.0045 (14)	−0.0141 (15)
C4A	0.0569 (14)	0.0553 (15)	0.0496 (15)	0.0052 (11)	0.0054 (11)	−0.0007 (12)
C4B	0.0530 (13)	0.0669 (16)	0.0498 (15)	−0.0043 (11)	0.0039 (11)	−0.0146 (13)
C5A	0.0463 (12)	0.0448 (13)	0.0457 (13)	−0.0011 (9)	0.0007 (10)	−0.0065 (11)
C5B	0.0420 (12)	0.0532 (14)	0.0487 (14)	0.0027 (10)	−0.0003 (10)	−0.0138 (11)
C6A	0.0624 (15)	0.0814 (19)	0.0753 (19)	−0.0104 (13)	0.0108 (14)	0.0039 (15)
C6B	0.0585 (15)	0.110 (2)	0.083 (2)	0.0064 (15)	0.0099 (14)	−0.0417 (18)
C7A	0.0594 (14)	0.0585 (16)	0.0539 (16)	−0.0055 (11)	−0.0152 (12)	−0.0061 (13)
C7B	0.0583 (14)	0.0611 (15)	0.0473 (15)	0.0093 (11)	−0.0104 (11)	−0.0069 (12)
C8A	0.0609 (15)	0.0619 (17)	0.0667 (17)	−0.0073 (13)	−0.0027 (13)	0.0076 (13)
C8B	0.0599 (15)	0.0656 (17)	0.0630 (17)	0.0120 (12)	−0.0042 (13)	−0.0188 (13)
C9A	0.0542 (15)	0.0771 (19)	0.086 (2)	−0.0043 (13)	0.0022 (14)	0.0000 (17)
C9B	0.0537 (14)	0.0768 (19)	0.0741 (19)	0.0055 (13)	0.0004 (13)	−0.0171 (15)
C10A	0.0649 (16)	0.0589 (17)	0.092 (2)	−0.0104 (13)	−0.0176 (15)	0.0027 (16)
C10B	0.0577 (15)	0.0664 (17)	0.084 (2)	0.0142 (13)	−0.0157 (14)	−0.0206 (16)
C11A	0.086 (2)	0.083 (2)	0.077 (2)	0.0012 (16)	−0.0144 (17)	0.0248 (16)
C11B	0.0786 (19)	0.103 (2)	0.070 (2)	0.0010 (17)	−0.0097 (16)	−0.0391 (17)
C12A	0.0778 (18)	0.097 (2)	0.0528 (17)	0.0059 (16)	−0.0081 (13)	0.0088 (16)
C12B	0.0719 (17)	0.107 (2)	0.0532 (17)	−0.0045 (16)	−0.0018 (13)	−0.0202 (16)
C13A	0.085 (2)	0.070 (2)	0.166 (3)	0.0032 (16)	−0.020 (2)	0.001 (2)
C13B	0.0794 (19)	0.082 (2)	0.136 (3)	0.0029 (16)	−0.0143 (18)	−0.040 (2)
C14A	0.0558 (14)	0.0444 (14)	0.0570 (16)	−0.0012 (11)	−0.0006 (12)	−0.0053 (12)
C14B	0.0526 (13)	0.0484 (14)	0.0578 (16)	−0.0002 (11)	0.0021 (11)	−0.0100 (12)
C15A	0.0652 (15)	0.0558 (16)	0.0557 (16)	−0.0081 (11)	−0.0087 (12)	0.0026 (13)
C15B	0.0632 (14)	0.0586 (15)	0.0526 (15)	0.0025 (11)	0.0003 (12)	−0.0186 (12)
C16A	0.111 (2)	0.0654 (19)	0.076 (2)	−0.0039 (16)	−0.0194 (17)	0.0155 (16)
C16B	0.100 (2)	0.103 (2)	0.0568 (18)	−0.0139 (18)	0.0126 (16)	−0.0108 (17)
C17A	0.110 (2)	0.123 (3)	0.060 (2)	−0.006 (2)	0.0074 (17)	−0.0110 (19)
C17B	0.106 (2)	0.075 (2)	0.0696 (19)	−0.0024 (16)	−0.0055 (16)	−0.0301 (16)
C18A	0.0457 (12)	0.0433 (13)	0.0487 (14)	−0.0048 (10)	0.0020 (10)	−0.0059 (11)
C18B	0.0434 (12)	0.0448 (13)	0.0523 (14)	0.0038 (10)	−0.0021 (10)	−0.0124 (11)
C19A	0.0549 (13)	0.0547 (15)	0.0610 (16)	0.0002 (11)	−0.0036 (12)	−0.0128 (13)
C19B	0.0542 (13)	0.0618 (16)	0.0622 (16)	0.0004 (11)	−0.0092 (12)	−0.0090 (14)
C20A	0.0701 (16)	0.0656 (17)	0.0629 (17)	−0.0116 (13)	−0.0086 (13)	−0.0184 (14)
C20B	0.0656 (16)	0.0656 (18)	0.0734 (19)	0.0127 (13)	−0.0210 (13)	0.0009 (15)
C21A	0.0795 (18)	0.0570 (16)	0.0638 (18)	−0.0051 (14)	0.0076 (14)	−0.0204 (13)
C21B	0.0747 (17)	0.0571 (17)	0.088 (2)	0.0014 (14)	−0.0151 (15)	0.0117 (15)
C22A	0.0673 (17)	0.0725 (19)	0.086 (2)	0.0148 (13)	0.0004 (15)	−0.0308 (16)
C22B	0.0638 (16)	0.0688 (19)	0.117 (3)	−0.0151 (13)	−0.0227 (16)	0.0273 (18)
C23A	0.0545 (14)	0.0678 (17)	0.0697 (18)	0.0053 (12)	−0.0036 (12)	−0.0196 (14)
C23B	0.0530 (14)	0.0607 (16)	0.0824 (19)	−0.0025 (12)	−0.0193 (13)	0.0092 (14)
C24A	0.133 (3)	0.085 (2)	0.102 (3)	0.0090 (19)	−0.001 (2)	−0.049 (2)
C24B	0.126 (3)	0.086 (2)	0.153 (3)	−0.016 (2)	−0.048 (2)	0.050 (2)
N1A	0.0550 (11)	0.0465 (11)	0.0467 (12)	−0.0049 (8)	−0.0083 (9)	−0.0045 (9)
N1B	0.0522 (11)	0.0483 (11)	0.0478 (12)	0.0059 (8)	−0.0029 (9)	−0.0092 (9)
O1A	0.1181 (16)	0.147 (2)	0.0728 (14)	0.0047 (14)	0.0338 (13)	−0.0354 (14)
O1B	0.1148 (16)	0.150 (2)	0.0722 (15)	−0.0259 (14)	0.0368 (13)	0.0034 (14)
O2A	0.0700 (11)	0.0646 (11)	0.0909 (14)	−0.0230 (9)	−0.0138 (9)	0.0031 (10)
O2B	0.0688 (11)	0.0805 (13)	0.0948 (14)	0.0267 (10)	−0.0071 (9)	−0.0332 (11)

### Geometric parameters (Å, º)

**Table:**

C1A—N1A	1.471 (3)	C13A—H13A	0.9600
C1A—C7A	1.523 (3)	C13A—H13B	0.9600
C1A—C2A	1.524 (3)	C13A—H13C	0.9600
C1A—H1A	0.9800	C13B—H13D	0.9600
C1B—N1B	1.476 (3)	C13B—H13E	0.9600
C1B—C7B	1.515 (3)	C13B—H13F	0.9600
C1B—C2B	1.523 (3)	C14A—O2A	1.233 (2)
C1B—H1B	0.9800	C14A—O2A	1.233 (2)
C2A—C3A	1.494 (3)	C14A—N1A	1.360 (3)
C2A—H2A1	0.9700	C14A—C15A	1.473 (3)
C2A—H2A2	0.9700	C14B—O2B	1.230 (2)
C2B—C3B	1.487 (3)	C14B—N1B	1.359 (3)
C2B—H2B1	0.9700	C14B—C15B	1.475 (3)
C2B—H2B2	0.9700	C15A—C16A	1.482 (3)
C3A—O1A	1.202 (3)	C15A—C17A	1.488 (3)
C3A—C4A	1.497 (3)	C15A—H15A	0.9800
C3B—O1B	1.206 (3)	C15B—C16B	1.493 (3)
C3B—C4B	1.506 (4)	C15B—C17B	1.495 (3)
C4A—C6A	1.521 (3)	C15B—H15B	0.9800
C4A—C5A	1.536 (3)	C16A—C17A	1.445 (4)
C4A—H4A	0.9800	C16A—H16A	0.9700
C4B—C6B	1.517 (3)	C16A—H16B	0.9700
C4B—C5B	1.536 (3)	C16B—C17B	1.460 (3)
C4B—H4B	0.9800	C16B—H16C	0.9700
C5A—N1A	1.479 (2)	C16B—H16D	0.9700
C5A—C18A	1.514 (3)	C17A—H17A	0.9700
C5A—H5A	0.9800	C17A—H17B	0.9700
C5B—N1B	1.484 (2)	C17B—H17C	0.9700
C5B—C18B	1.512 (3)	C17B—H17D	0.9700
C5B—H5B	0.9800	C18A—C19A	1.370 (3)
C6A—H6A1	0.9600	C18A—C23A	1.380 (3)
C6A—H6A2	0.9600	C18B—C19B	1.368 (3)
C6A—H6A3	0.9600	C18B—C23B	1.368 (3)
C6B—H6B1	0.9600	C19A—C20A	1.384 (3)
C6B—H6B2	0.9600	C19A—H19A	0.9300
C6B—H6B3	0.9600	C19B—C20B	1.380 (3)
C7A—C12A	1.375 (3)	C19B—H19B	0.9300
C7A—C8A	1.382 (3)	C20A—C21A	1.371 (3)
C7B—C12B	1.366 (3)	C20A—H20A	0.9300
C7B—C8B	1.382 (3)	C20B—C21B	1.370 (3)
C8A—C9A	1.379 (3)	C20B—H20B	0.9300
C8A—H8A	0.9300	C21A—C22A	1.365 (4)
C8B—C9B	1.379 (3)	C21A—C24A	1.510 (3)
C8B—H8B	0.9300	C21B—C22B	1.367 (4)
C9A—C10A	1.372 (4)	C21B—C24B	1.518 (4)
C9A—H9A	0.9300	C22A—C23A	1.386 (3)
C9B—C10B	1.374 (3)	C22A—H22A	0.9300
C9B—H9B	0.9300	C22B—C23B	1.380 (3)
C10A—C11A	1.369 (3)	C22B—H22B	0.9300
C10A—C13A	1.513 (4)	C23A—H23A	0.9300
C10B—C11B	1.374 (3)	C23B—H23B	0.9300
C10B—C13B	1.507 (3)	C24A—H24A	0.9600
C11A—C12A	1.376 (3)	C24A—H24B	0.9600
C11A—H11A	0.9300	C24A—H24C	0.9600
C11B—C12B	1.380 (4)	C24B—H24D	0.9600
C11B—H11B	0.9300	C24B—H24E	0.9600
C12A—H12A	0.9300	C24B—H24F	0.9600
C12B—H12B	0.9300		
			
N1A—C1A—C7A	112.45 (18)	H13A—C13A—H13C	109.5
N1A—C1A—C2A	108.28 (18)	H13B—C13A—H13C	109.5
C7A—C1A—C2A	116.9 (2)	C10B—C13B—H13D	109.5
N1A—C1A—H1A	106.2	C10B—C13B—H13E	109.5
C7A—C1A—H1A	106.2	H13D—C13B—H13E	109.5
C2A—C1A—H1A	106.2	C10B—C13B—H13F	109.5
N1B—C1B—C7B	112.72 (19)	H13D—C13B—H13F	109.5
N1B—C1B—C2B	107.62 (18)	H13E—C13B—H13F	109.5
C7B—C1B—C2B	117.1 (2)	O2A—C14A—N1A	121.2 (2)
N1B—C1B—H1B	106.2	O2A—C14A—N1A	121.2 (2)
C7B—C1B—H1B	106.2	O2A—C14A—C15A	119.4 (2)
C2B—C1B—H1B	106.2	O2A—C14A—C15A	119.4 (2)
C3A—C2A—C1A	114.4 (2)	N1A—C14A—C15A	119.3 (2)
C3A—C2A—H2A1	108.7	O2B—C14B—N1B	121.9 (2)
C1A—C2A—H2A1	108.7	O2B—C14B—C15B	119.8 (2)
C3A—C2A—H2A2	108.7	N1B—C14B—C15B	118.29 (19)
C1A—C2A—H2A2	108.7	C14A—C15A—C16A	117.1 (2)
H2A1—C2A—H2A2	107.6	C14A—C15A—C17A	117.6 (2)
C3B—C2B—C1B	114.3 (2)	C16A—C15A—C17A	58.24 (17)
C3B—C2B—H2B1	108.7	C14A—C15A—H15A	117.0
C1B—C2B—H2B1	108.7	C16A—C15A—H15A	117.0
C3B—C2B—H2B2	108.7	C17A—C15A—H15A	117.0
C1B—C2B—H2B2	108.7	C14B—C15B—C16B	117.8 (2)
H2B1—C2B—H2B2	107.6	C14B—C15B—C17B	116.7 (2)
O1A—C3A—C2A	121.0 (2)	C16B—C15B—C17B	58.50 (16)
O1A—C3A—C4A	123.0 (3)	C14B—C15B—H15B	117.0
C2A—C3A—C4A	116.00 (19)	C16B—C15B—H15B	117.0
O1B—C3B—C2B	120.9 (3)	C17B—C15B—H15B	117.0
O1B—C3B—C4B	122.6 (3)	C17A—C16A—C15A	61.10 (18)
C2B—C3B—C4B	116.46 (19)	C17A—C16A—H16A	117.7
C3A—C4A—C6A	112.08 (19)	C15A—C16A—H16A	117.7
C3A—C4A—C5A	111.48 (18)	C17A—C16A—H16B	117.7
C6A—C4A—C5A	111.3 (2)	C15A—C16A—H16B	117.7
C3A—C4A—H4A	107.2	H16A—C16A—H16B	114.8
C6A—C4A—H4A	107.2	C17B—C16B—C15B	60.81 (16)
C5A—C4A—H4A	107.2	C17B—C16B—H16C	117.7
C3B—C4B—C6B	112.18 (19)	C15B—C16B—H16C	117.7
C3B—C4B—C5B	111.23 (19)	C17B—C16B—H16D	117.7
C6B—C4B—C5B	111.4 (2)	C15B—C16B—H16D	117.7
C3B—C4B—H4B	107.2	H16C—C16B—H16D	114.8
C6B—C4B—H4B	107.2	C16A—C17A—C15A	60.66 (17)
C5B—C4B—H4B	107.2	C16A—C17A—H17A	117.7
N1A—C5A—C18A	112.76 (15)	C15A—C17A—H17A	117.7
N1A—C5A—C4A	111.62 (18)	C16A—C17A—H17B	117.7
C18A—C5A—C4A	110.06 (17)	C15A—C17A—H17B	117.7
N1A—C5A—H5A	107.4	H17A—C17A—H17B	114.8
C18A—C5A—H5A	107.4	C16B—C17B—C15B	60.69 (16)
C4A—C5A—H5A	107.4	C16B—C17B—H17C	117.7
N1B—C5B—C18B	112.55 (15)	C15B—C17B—H17C	117.7
N1B—C5B—C4B	111.12 (17)	C16B—C17B—H17D	117.7
C18B—C5B—C4B	110.95 (18)	C15B—C17B—H17D	117.7
N1B—C5B—H5B	107.3	H17C—C17B—H17D	114.8
C18B—C5B—H5B	107.3	C19A—C18A—C23A	117.8 (2)
C4B—C5B—H5B	107.3	C19A—C18A—C5A	120.37 (19)
C4A—C6A—H6A1	109.5	C23A—C18A—C5A	121.8 (2)
C4A—C6A—H6A2	109.5	C19B—C18B—C23B	117.5 (2)
H6A1—C6A—H6A2	109.5	C19B—C18B—C5B	120.83 (19)
C4A—C6A—H6A3	109.5	C23B—C18B—C5B	121.7 (2)
H6A1—C6A—H6A3	109.5	C18A—C19A—C20A	120.7 (2)
H6A2—C6A—H6A3	109.5	C18A—C19A—H19A	119.6
C4B—C6B—H6B1	109.5	C20A—C19A—H19A	119.6
C4B—C6B—H6B2	109.5	C18B—C19B—C20B	121.1 (2)
H6B1—C6B—H6B2	109.5	C18B—C19B—H19B	119.4
C4B—C6B—H6B3	109.5	C20B—C19B—H19B	119.4
H6B1—C6B—H6B3	109.5	C21A—C20A—C19A	121.9 (2)
H6B2—C6B—H6B3	109.5	C21A—C20A—H20A	119.1
C12A—C7A—C8A	117.0 (2)	C19A—C20A—H20A	119.1
C12A—C7A—C1A	124.8 (2)	C21B—C20B—C19B	121.8 (2)
C8A—C7A—C1A	118.3 (2)	C21B—C20B—H20B	119.1
C12B—C7B—C8B	116.8 (2)	C19B—C20B—H20B	119.1
C12B—C7B—C1B	124.4 (2)	C22A—C21A—C20A	117.2 (2)
C8B—C7B—C1B	118.8 (2)	C22A—C21A—C24A	121.5 (3)
C9A—C8A—C7A	120.9 (2)	C20A—C21A—C24A	121.3 (3)
C9A—C8A—H8A	119.5	C22B—C21B—C20B	116.7 (2)
C7A—C8A—H8A	119.5	C22B—C21B—C24B	121.3 (3)
C9B—C8B—C7B	121.8 (2)	C20B—C21B—C24B	122.0 (3)
C9B—C8B—H8B	119.1	C21A—C22A—C23A	121.7 (2)
C7B—C8B—H8B	119.1	C21A—C22A—H22A	119.1
C10A—C9A—C8A	122.2 (2)	C23A—C22A—H22A	119.1
C10A—C9A—H9A	118.9	C21B—C22B—C23B	121.9 (2)
C8A—C9A—H9A	118.9	C21B—C22B—H22B	119.1
C10B—C9B—C8B	121.2 (2)	C23B—C22B—H22B	119.1
C10B—C9B—H9B	119.4	C18A—C23A—C22A	120.7 (2)
C8B—C9B—H9B	119.4	C18A—C23A—H23A	119.6
C11A—C10A—C9A	116.4 (3)	C22A—C23A—H23A	119.6
C11A—C10A—C13A	121.4 (3)	C18B—C23B—C22B	121.1 (2)
C9A—C10A—C13A	122.1 (3)	C18B—C23B—H23B	119.5
C11B—C10B—C9B	116.9 (2)	C22B—C23B—H23B	119.5
C11B—C10B—C13B	121.4 (3)	C21A—C24A—H24A	109.5
C9B—C10B—C13B	121.7 (2)	C21A—C24A—H24B	109.5
C10A—C11A—C12A	122.2 (3)	H24A—C24A—H24B	109.5
C10A—C11A—H11A	118.9	C21A—C24A—H24C	109.5
C12A—C11A—H11A	118.9	H24A—C24A—H24C	109.5
C10B—C11B—C12B	121.8 (3)	H24B—C24A—H24C	109.5
C10B—C11B—H11B	119.1	C21B—C24B—H24D	109.5
C12B—C11B—H11B	119.1	C21B—C24B—H24E	109.5
C7A—C12A—C11A	121.2 (2)	H24D—C24B—H24E	109.5
C7A—C12A—H12A	119.4	C21B—C24B—H24F	109.5
C11A—C12A—H12A	119.4	H24D—C24B—H24F	109.5
C7B—C12B—C11B	121.5 (2)	H24E—C24B—H24F	109.5
C7B—C12B—H12B	119.2	C14A—N1A—C1A	117.82 (18)
C11B—C12B—H12B	119.2	C14A—N1A—C5A	121.97 (19)
C10A—C13A—H13A	109.5	C1A—N1A—C5A	119.75 (17)
C10A—C13A—H13B	109.5	C14B—N1B—C1B	117.74 (18)
H13A—C13A—H13B	109.5	C14B—N1B—C5B	121.91 (19)
C10A—C13A—H13C	109.5	C1B—N1B—C5B	120.12 (16)
			
N1A—C1A—C2A—C3A	47.4 (3)	O2B—C14B—C15B—C17B	25.2 (3)
C7A—C1A—C2A—C3A	−80.8 (3)	N1B—C14B—C15B—C17B	−153.8 (2)
N1B—C1B—C2B—C3B	−47.6 (3)	C14A—C15A—C16A—C17A	107.2 (2)
C7B—C1B—C2B—C3B	80.6 (3)	C14B—C15B—C16B—C17B	105.9 (3)
C1A—C2A—C3A—O1A	−179.8 (2)	C14A—C15A—C17A—C16A	−106.3 (3)
C1A—C2A—C3A—C4A	−0.4 (3)	C14B—C15B—C17B—C16B	−107.6 (2)
C1B—C2B—C3B—O1B	179.8 (2)	N1A—C5A—C18A—C19A	−129.1 (2)
C1B—C2B—C3B—C4B	0.3 (3)	C4A—C5A—C18A—C19A	105.6 (2)
O1A—C3A—C4A—C6A	7.1 (4)	N1A—C5A—C18A—C23A	54.4 (3)
C2A—C3A—C4A—C6A	−172.2 (2)	C4A—C5A—C18A—C23A	−70.9 (2)
O1A—C3A—C4A—C5A	132.6 (3)	N1B—C5B—C18B—C19B	130.0 (2)
C2A—C3A—C4A—C5A	−46.7 (3)	C4B—C5B—C18B—C19B	−104.7 (2)
O1B—C3B—C4B—C6B	−6.8 (4)	N1B—C5B—C18B—C23B	−52.0 (3)
C2B—C3B—C4B—C6B	172.7 (2)	C4B—C5B—C18B—C23B	73.2 (2)
O1B—C3B—C4B—C5B	−132.4 (3)	C23A—C18A—C19A—C20A	0.6 (3)
C2B—C3B—C4B—C5B	47.1 (3)	C5A—C18A—C19A—C20A	−176.07 (19)
C3A—C4A—C5A—N1A	44.4 (2)	C23B—C18B—C19B—C20B	0.5 (3)
C6A—C4A—C5A—N1A	170.28 (17)	C5B—C18B—C19B—C20B	178.6 (2)
C3A—C4A—C5A—C18A	170.35 (18)	C18A—C19A—C20A—C21A	0.4 (4)
C6A—C4A—C5A—C18A	−63.7 (2)	C18B—C19B—C20B—C21B	−0.1 (4)
C3B—C4B—C5B—N1B	−43.7 (2)	C19A—C20A—C21A—C22A	−1.2 (4)
C6B—C4B—C5B—N1B	−169.65 (18)	C19A—C20A—C21A—C24A	178.2 (2)
C3B—C4B—C5B—C18B	−169.69 (18)	C19B—C20B—C21B—C22B	−0.5 (4)
C6B—C4B—C5B—C18B	64.3 (2)	C19B—C20B—C21B—C24B	178.6 (3)
N1A—C1A—C7A—C12A	−118.7 (3)	C20A—C21A—C22A—C23A	1.1 (4)
C2A—C1A—C7A—C12A	7.4 (4)	C24A—C21A—C22A—C23A	−178.3 (2)
N1A—C1A—C7A—C8A	61.2 (3)	C20B—C21B—C22B—C23B	0.8 (5)
C2A—C1A—C7A—C8A	−172.7 (2)	C24B—C21B—C22B—C23B	−178.4 (3)
N1B—C1B—C7B—C12B	118.2 (3)	C19A—C18A—C23A—C22A	−0.7 (3)
C2B—C1B—C7B—C12B	−7.5 (3)	C5A—C18A—C23A—C22A	175.9 (2)
N1B—C1B—C7B—C8B	−61.9 (3)	C21A—C22A—C23A—C18A	−0.1 (4)
C2B—C1B—C7B—C8B	172.4 (2)	C19B—C18B—C23B—C22B	−0.2 (4)
C12A—C7A—C8A—C9A	1.1 (4)	C5B—C18B—C23B—C22B	−178.3 (2)
C1A—C7A—C8A—C9A	−178.8 (2)	C21B—C22B—C23B—C18B	−0.4 (4)
C12B—C7B—C8B—C9B	−1.8 (4)	O2A—C14A—N1A—C1A	9.3 (3)
C1B—C7B—C8B—C9B	178.3 (2)	O2A—C14A—N1A—C1A	9.3 (3)
C7A—C8A—C9A—C10A	0.9 (4)	C15A—C14A—N1A—C1A	−169.75 (18)
C7B—C8B—C9B—C10B	−0.3 (4)	O2A—C14A—N1A—C5A	−178.56 (18)
C8A—C9A—C10A—C11A	−1.9 (4)	O2A—C14A—N1A—C5A	−178.56 (18)
C8A—C9A—C10A—C13A	177.3 (3)	C15A—C14A—N1A—C5A	2.4 (3)
C8B—C9B—C10B—C11B	2.0 (4)	C7A—C1A—N1A—C14A	−107.7 (2)
C8B—C9B—C10B—C13B	−177.4 (2)	C2A—C1A—N1A—C14A	121.7 (2)
C9A—C10A—C11A—C12A	1.0 (4)	C7A—C1A—N1A—C5A	80.0 (2)
C13A—C10A—C11A—C12A	−178.2 (3)	C2A—C1A—N1A—C5A	−50.7 (3)
C9B—C10B—C11B—C12B	−1.5 (4)	C18A—C5A—N1A—C14A	68.3 (2)
C13B—C10B—C11B—C12B	177.9 (3)	C4A—C5A—N1A—C14A	−167.24 (17)
C8A—C7A—C12A—C11A	−2.0 (4)	C18A—C5A—N1A—C1A	−119.7 (2)
C1A—C7A—C12A—C11A	177.9 (2)	C4A—C5A—N1A—C1A	4.8 (2)
C10A—C11A—C12A—C7A	1.0 (5)	O2B—C14B—N1B—C1B	−10.3 (3)
C8B—C7B—C12B—C11B	2.3 (4)	C15B—C14B—N1B—C1B	168.65 (19)
C1B—C7B—C12B—C11B	−177.8 (2)	O2B—C14B—N1B—C5B	175.21 (19)
C10B—C11B—C12B—C7B	−0.7 (4)	C15B—C14B—N1B—C5B	−5.8 (3)
O2A—C14A—C15A—C16A	−26.0 (3)	C7B—C1B—N1B—C14B	106.7 (2)
O2A—C14A—C15A—C16A	−26.0 (3)	C2B—C1B—N1B—C14B	−122.6 (2)
N1A—C14A—C15A—C16A	153.0 (2)	C7B—C1B—N1B—C5B	−78.8 (2)
O2A—C14A—C15A—C17A	40.4 (3)	C2B—C1B—N1B—C5B	51.9 (3)
O2A—C14A—C15A—C17A	40.4 (3)	C18B—C5B—N1B—C14B	−66.5 (2)
N1A—C14A—C15A—C17A	−140.5 (2)	C4B—C5B—N1B—C14B	168.39 (19)
O2B—C14B—C15B—C16B	−41.4 (3)	C18B—C5B—N1B—C1B	119.2 (2)
N1B—C14B—C15B—C16B	139.6 (2)	C4B—C5B—N1B—C1B	−5.9 (3)

### Hydrogen-bond geometry (Å, º)

Cg1 and Cg2 are the centroids of the C7A–C12A and C7B–C12B rings, respectively.

**Table:**

*D*—H···*A*	*D*—H	H···*A*	*D*···*A*	*D*—H···*A*
C23*A*—H23*A*···*Cg*1	0.93	2.86	3.787 (3)	173
C23*B*—H23*B*···*Cg*2	0.93	2.85	3.777 (3)	173
C5*A*—H5*A*···O2*B*^i^	0.98	2.41	3.366 (3)	166
C5*B*—H5*B*···O2*A*	0.98	2.42	3.381 (3)	166
C19*A*—H19*A*···O2*B*^i^	0.93	2.59	3.435 (3)	151

Symmetry code: (i) *x*−1, *y*, *z*.
