# Supplementary figures and images for: Crystal structure of 1-cyclo­propane­carbon­yl-3-methyl-2,6-di-p-tolyl­piperidin-4-one
Source: Acta Crystallogr Sect E Struct Rep Online. 2014 Aug 30;70(Pt 9):o1056–7. doi: 10.1107/S1600536814018546 (PMC4186191; doi:10.1107/S1600536814018546)

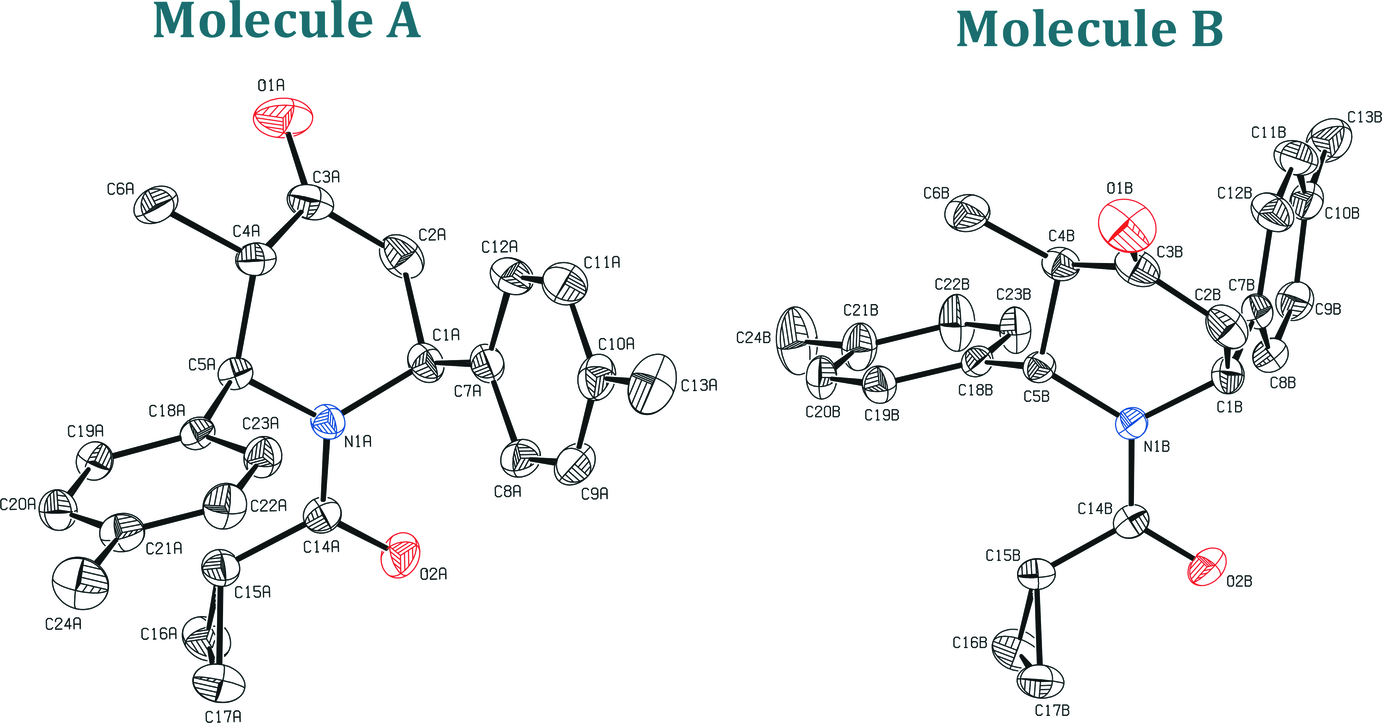

Supplement: Supplementary file 4 [file e-70-o1056-fig1.tif]

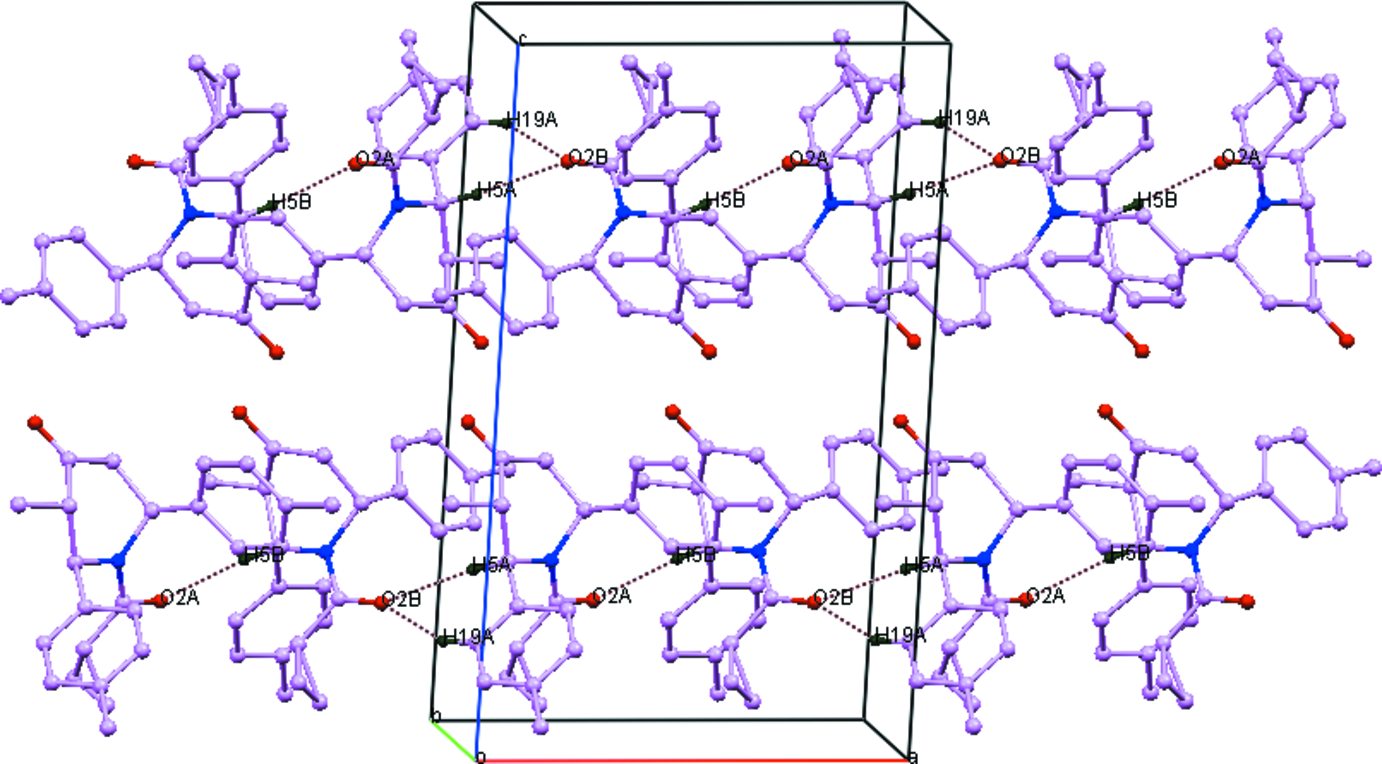

Supplement: Supplementary file 5 [file e-70-o1056-fig2.tif]
